# Unique Genetic Clades and Frequent Zoonotic Transmission of *Sporothrix brasiliensis* with Reduced Itraconazole Susceptibility

**DOI:** 10.1007/s11046-026-01101-5

**Published:** 2026-09-15

**Authors:** Mariana Rodrigues Trápaga, Bram Spruijtenburg, Jéssica Estefania Dávila Hidalgo, Bruna Jacomel, Isabel Martins Madrid, Daniela Isabel Brayer Pereira, Antonella Souza Mattei, Angelita Reis Gomes, Renata Osório de Faria, Flávio Silveira, Cecília Bittencourt Severo, Valério Rodrigues Aquino, Ana Paula Giolo Franz, Sonia de Ávila Botton, Rosely Maria Zancopé-Oliveira, Vanice Rodrigues Poester, Theun de Groot, Eelco F. J. Meijer, Melissa Orzechowski Xavier

**Affiliations:** 1 Postgraduate Program in Health Sciences, Faculty of Medicine, Federal University of Rio Grande (FAMED-FURG), Rio Grande, RS Brazil; 2 Mycology Laboratory, FAMED-FURG, Rio Grande, RS Brazil; 3 Department of Medical Microbiology, Radboudumc, Nijmegen, The Netherlands; 4 Department of Medical Microbiology and Immunology, Canisius-Wilhelmina Hospital (CWZ)/Dicoon, Nijmegen, The Netherlands; 5 Radboudumc-CWZ Center of Expertise for Mycology, Nijmegen, The Netherlands; 6 Postgraduate Program in Microbiology, Parasitology and Pathology, Department of Basic Pathology, Biological Sciences, Federal University of Paraná, Curitiba, Brazil; 7 Zoonosis Control Center From the Municipality of Pelotas, Pelotas, RS Brazil; 8 Postgraduate Program in Microbiology and Parasitology, Mycology Laboratory, Department of Microbiology and Parasitology, Institute of Biology, Federal University of Pelotas (UFPel), Pelotas, RS Brazil; 9 University of Caxias Do Sul (UCS), Caxias Do Sul, RS Brazil; 10 Department of Preventive Veterinary Medicine, Faculty of Veterinary Medicine, Federal University of Pelotas (UFPel), Pelotas, RS Brazil; 11 Department of Agricultural Diagnosis and Research (DDPA), Secretariat of Agriculture (SEAPI), Desidério Finamor Veterinary Research Institute (IPVDF), Eldorado Do Sul, RS Brazil; 12 Federal University of Health Sciences of Porto Alegre (UFCSPA), Porto Alegre, RS Brazil; 13 Hospital de Clinicas de Porto Alegre, (HCPA), Porto Alegre, Brazil; 14 Clinical Analysis Laboratory, Hospital de Clínicas de Passo Fundo, Passo Fundo, RS Brazil; 15 Postgraduate Program in Pharmaceutical Sciences (PPGCF) and Veterinary Medicine (PPGMV), Center of Rural Sciences, Federal University of Santa Maria (UFSM), Santa Maria, RS Brazil; 16 Mycology Laboratory, Evandro Chagas National Institute of Infectology, Oswaldo Cruz Foundation (INI/Fiocruz), Rio de Janeiro, Brazil

**Keywords:** Antifungal resistance, Itraconazole, *Sporothrix brasiliensis*, Sporotrichosis, Genetic epidemiology

## Abstract

*Sporothrix brasiliensis* is driving a major zoonotic epidemic in Brazil, predominantly via cat-to-human transmission. In the late 1990s major outbreaks were reported in Rio de Janeiro (RJ) and Rio Grande do Sul (RS), the southernmost state. We characterized the antifungal susceptibility and genetic diversity of 450 *S. brasiliensis* isolates collected between 2009 and 2024 in RS to better understand treatment response, emergence and transmission.

A bimodal distribution of the itraconazole minimum inhibitory concentration (MIC) was observed with 24% non-wild-type (NWT) isolates. Short tandem repeat genotyping revealed three main clades: One associated with RJ strains (Clade I) and two novel clades (Clades II and III). Clade II formed large transmission clusters with < 20% of isolates being NWT for itraconazole, similar to Clade I. Clade III demonstrated less clustering, higher genetic variation and NWT MICs for 89% of isolates. Finally, all NWT strains in human patients with cat-transmitted disease and available clinical data, appeared isolated prior to azole exposure.

We report the emergence of genetically distinct *S. brasiliensis* clades in RS with different rates of reduced itraconazole susceptibility and evidence of zoonotic transmission of NWT isolates. Our findings highlight the urgent need for antifungal stewardship and surveillance in these endemic regions.

**Supplementary Information:**

The online version contains supplementary material available at https://doi.org/10.1007/s11046-026-01101-5.

## Introduction

Sporotrichosis is the most common type of subcutaneous mycosis worldwide and is considered a neglected tropical disease by the World Health Organization [1]. In the last decades, there has been a dramatic increase in sporotrichosis cases in Brazil. This is caused by the sudden emergence and spread of *Sporothrix brasiliensis*, a previously unrecognized species [2]. This fungus first emerged in cats, which develop severe disease and became the main host and source of infection. *S. brasiliensis* has rapidly spread mostly via inoculation by cats (scratching, biting), currently affecting thousands of felines, dogs and humans every year, with numbers still increasing [3]. This cat-transmitted sporotrichosis also led to outbreaks in other South American countries, with some cases reported outside the continent [4].

Itraconazole is the first-line therapy for all hosts without contraindications (e.g. pregnancy, drug interaction) [5, 6]. Via oral tablets or suspensions patients are treated daily for a prolonged duration of, usually, at least four months. This treatment, especially in cats, poses significant challenges. In domestic cats, the main challenges are the severity of the clinical presentation, the costs and prolonged treatment duration, and the reliance on owner compliance [7]. These factors contribute to a treatment abandonment rate of approximately 30–40% and cure rates ranging from 50 to 72% [5, 8]. In humans, treatment is generally more successful. Nonetheless, there have been reports of undesirable side-effects and reduced in vitro susceptibility of *S. brasiliensis* to itraconazole, highlighting the need for ongoing monitoring and studies to investigate if this is associated with clinical failure or a specific genetic background [9, 10]. The main strategies to tackle endemic zoonotic sporotrichosis include early diagnosis and effective treatment, but also robust epidemiological surveillance. The latter offers insights into sources of transmission and infection dynamics, allowing public health policies to control and manage outbreaks. In this context, human sporotrichosis became a notifiable disease in Brazil in 2025 [11].

In the late 1990s the first cat-transmitted sporotrichosis cases in Brazil were reported from the non-neighbouring states Rio de Janeiro and Rio Grande do Sul, which was followed by enormous outbreaks in both states [12, 13]. Although these outbreaks began around the same time, the oldest isolates from each region were genetically distinct, suggesting independent introductions [14–17]. Isolates highly related to the Rio de Janeiro genotype were also found in many other Brazilian states. The massive dispersal of isolates from the Rio de Janeiro associated clade indicates a huge dissemination potential. As only few isolates from other states were related to isolates from Rio Grande do Sul, it is still unknown whether the outbreak of *S. brasiliensis* in Rio Grande do Sul also involved clonal transmission from a potential local clade or whether it involved various independent introductions, possibly combined with spread from the Rio de Janeiro associated clade [14, 16]. Remarkably, epidemiological studies in Rio Grande do Sul have shown that a higher dose of itraconazole is needed to achieve clinical cure compared to cases in Rio de Janeiro (200 mg/day vs. 100 mg/day) [6, 18], suggesting genetic differences play a role.

This study aimed to understand the unique sporotrichosis situation in Rio Grande do Sul in which massive outbreaks are combined with seemingly reduced antifungal susceptibility. For this purpose, we evaluated antifungal susceptibility profiles and genetic diversity of *S. brasiliensis* isolates from Rio Grande do Sul and consequent impacts on the zoonotic transmission of this fungus.

## Materials and Methods

### Isolates

*S. brasiliensis* strains, collected in a period of 15 years (from 2009 to 2024), were isolated from clinical samples from cats, humans, dogs, cattle and from the environment. Isolates were obtained from laboratory culture collections from different Rio Grande do Sul health district regions, including the South (Federal University of Rio Grande, Federal University of Pelotas, Zoonosis Control Center of Pelotas), Metropolitan (Federal University of Health Sciences of Porto Alegre, Porto Alegre Clinical Hospital, State Center for Animal Health Diagnosis and Research), Sierra (University of Caxias do Sul), North (Passo Fundo Clinical Hospital) and Midwest (Federal University of Santa Maria) districts.

From the host institution, the Federal University of Rio Grande, *Sporothrix* isolates were randomly selected from the large culture collection (more than 4,000 isolates) to represent different host species and years of isolation. More recent years are proportionally more represented, reflecting the increasing number of sporotrichosis cases and isolate availability over time. Isolates from the remaining collaborating centers were included according to culture availability, aiming to represent the different geographic regions of Rio Grande do Sul.

The study was performed using archived fungal isolates obtained from microbiological culture collections, without access to identifiable patient information. Therefore ethical committee approval was not required. The transfer and research use of isolates obtained from collaborating institutions were authorized through institutional material transfer agreements (MTAs) or equivalent authorization documents.

### Antifungal Susceptibility Testing (AFST)

The antifungal susceptibility profiles of *S. brasiliensis* in the mould phase were evaluated by broth microdilution assay according to the Clinical and Laboratory Standards Institute (CLSI) M38-A2 guideline [19]. The isolates were cultured in Potato Dextrose Agar for 7 days at 30 °C and the inoculum was standardized by spectrophotometry in an interval of 0.8 × 10^4^ to 10^5^ CFU/mL. The drugs itraconazole (Janssen Cilag, Breda, The Netherlands), posaconazole (Merck, Darmstadt, Germany), isavuconazole (Brasilea Pharmaceutica, Basel, Switzerland), voriconazole (Pfizer Central Research, Sandwich, United Kingdom) and amphotericin B (Bristol Myers Squib, Woerden, The Netherlands) were tested in the final concentrations between 0.016 and 16 µg/mL. Fluconazole (Merck, Darmstadt, Germany), was tested in the final concentrations of 0.063 to 64 µg/mL. Terbinafine (Novartis Pharmaceuticals, Basel, Switzerland) was tested on itraconazole non-wild-type (NWT) isolates between the concentrations 0.004 and 4 µg/mL.

For all antifungals the minimal inhibitory concentration (MIC) was visually determined. For azoles and amphotericin B the MIC was defined as the lowest concentration inhibiting 100% of fungal growth, while for terbinafine this was 80% of fungal growth. Isolates were classified as wild-type (WT) and non-wild-type (NWT) according to the most recently established epidemiological cut-off values (ECVs) of 8 µg/mL for itraconazole, 4 µg/mL for posaconazole and amphotericin B and 0.125 µg/mL for terbinafine [20]. For itraconazole, NWT isolates were classified as exhibiting either unaffected or partially inhibited growth compared to the growth control, corresponding to no inhibition or incomplete (non-100%) inhibition, respectively. To analyse the antifungal susceptibility profile of the isolates, we used the geometric mean, and MIC 50% and 90%, which indicate the concentration able to inhibit 50% and 90% of the isolates, respectively.

### Genotyping by STR

Genotyping was performed using the previously developed short tandem repeat (STR) analysis as described earlier [21]. DNA was extracted by dissolving cells in 50 µL of MightyPrep Reagent for DNA (Takara Bio Inc., Shiga, Japan). Next, three multiplex polymerase chain reactions (PCRs), targeting tri- and hexanucleotide repeats (M3-I, M3-II, M6) were used to amplify the microsatellite markers. Each PCR reaction consisted of 1× Terra PCR Direct Buffer (Takara Bio Inc.), forward and reverse primers (0.1 to 1 µM), 12.5 µL sterile water, 1.25 U Terra PCR Direct Polymerase mix and 1.5 µL of DNA. Amplicons were diluted 1000 times and 0.12 µL Orange-600 DNA Size Standard (NimaGen, Nijmegen, The Netherlands), followed by capillary electrophoresis on a 3500XL genetic analyser (Applied Biosystems, Foster City, CA, USA) [21]. Copy numbers were determined using GeneMapper5 (Applied Biosystems, Foster City, CA, USA) and the genetic analysis were done with BioNumerics v7.6.1 (Apllied Maths NV, Sint-Martems-Latem, Belgium). The current found genotypes were compared with all previously genotyped isolates [22].

### Statistical Analysis

MIC data were analysed by dividing the isolates into groups according to the WT and NWT classification for itraconazole, posaconazole and amphotericin B, the geographic origin based on the health district of Rio Grande do Sul, the genetic clade determined by STR results, and the period of isolation (2009–2014; 2015–2019; 2020–2024). Analyses were conducted using descriptive and frequency tests to compare groups. The Chi-square test was used to compare the Rio Grande do Sul health district and WT or NWT (growth inhibition or unaffected growth) for itraconazole; the clade and WT or NWT (growth inhibition or unaffected growth) for itraconazole; the host and WT or NWT for itraconazole; and the period and WT or NWT for itraconazole. All tests were performed using the SPSS 20.0 statistical program (IBM, Chicago, IL, US).

## Results

### Epidemiological Data

**Fig. 1:**
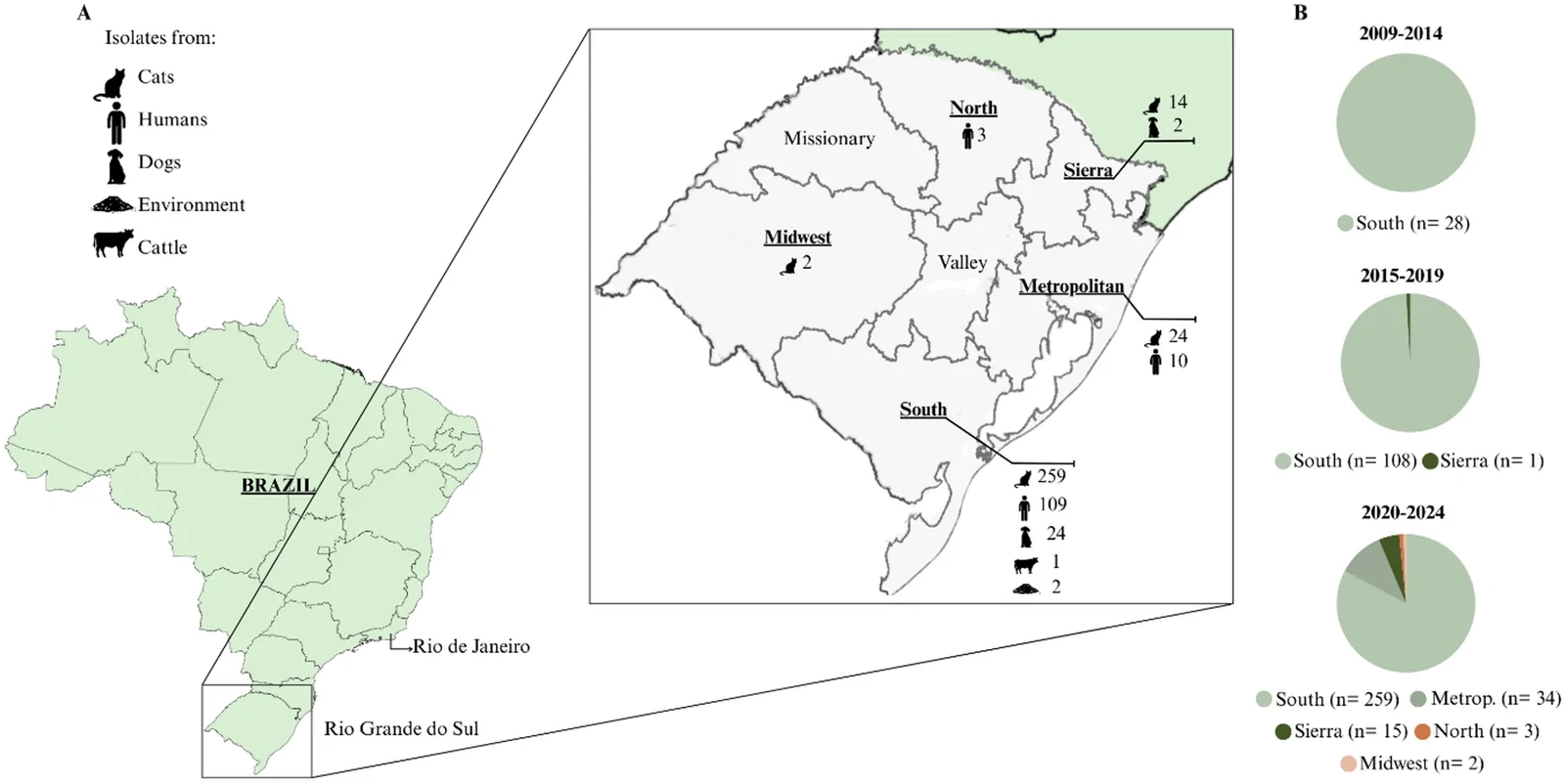
Origin of *Sporothrix brasiliensis* isolates. **A** Map of Brazil with indicated Rio Grande do Sul and Rio de Janeiro states (left panel) and health districts of Rio Grande do Sul (right panel). Numbers at icons indicate the number of isolates obtained from each host or source. **B** Collection period of isolates from Rio Grande do Sul, categorized by health district regions

From 2009 to 2024, a total of 450 *S. brasiliensis* isolates was collected from cats (n=299), humans (n=122), dogs (n=26), the environment (n=2) and cattle (n=1) in the state of Rio Grande do Sul, Brazil. The environmental isolates were collected from soil in areas with presence of cats with sporotrichosis. Isolates were collected from different areas of this state, of which the majority originated from the South health district region (n=395), while other isolates came from Metropolitan (n=34), Sierra (n=16), North (n=3) and Midwest (n=2) (Fig. 1A). From all isolates 28 were collected in 2009–2014, 109 in 2015–2019, and 313 in 2020–2024 (Fig. 1B).

### Antifungal Susceptibility Profiles

**Fig. 2:**
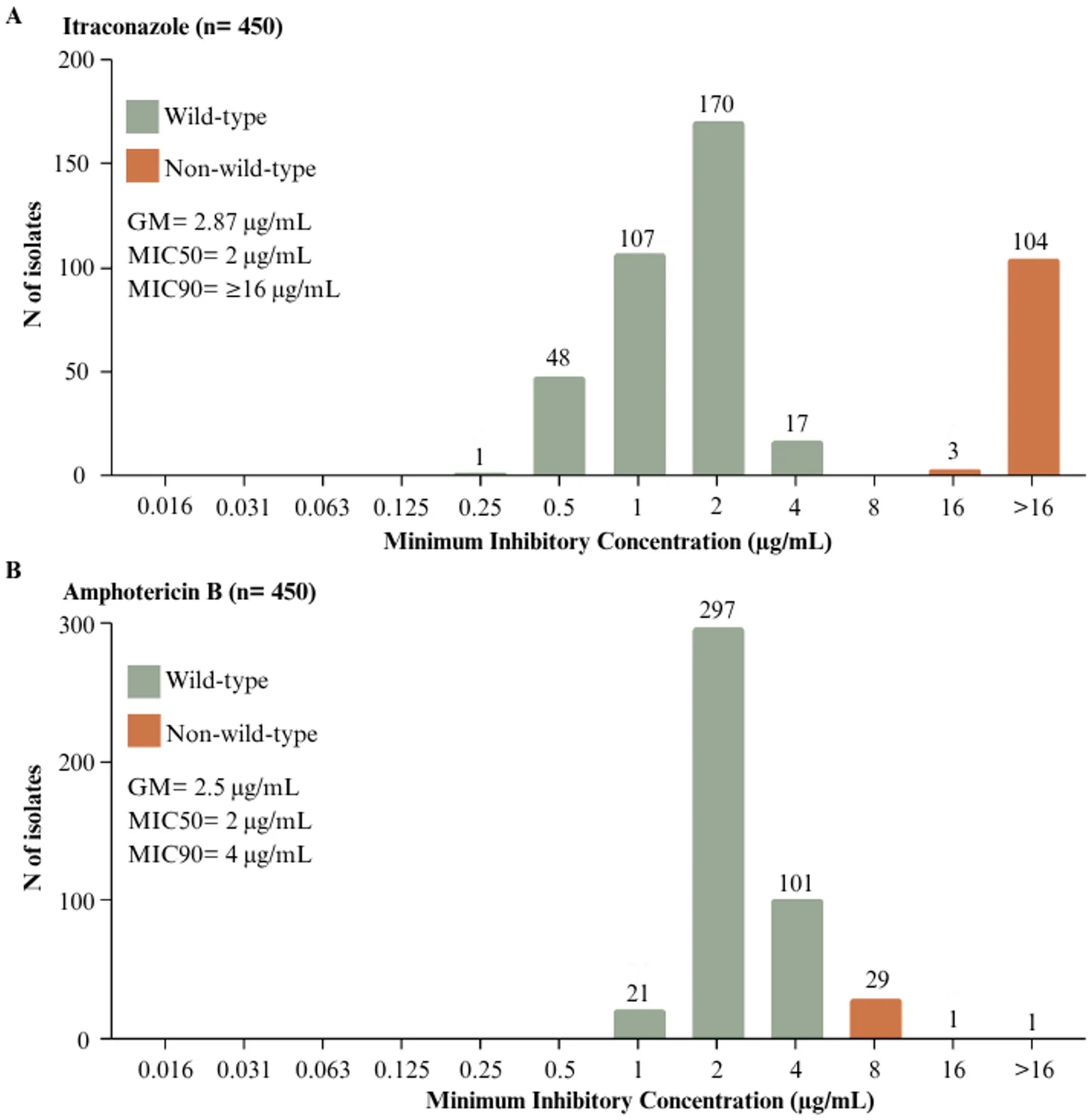
Distribution of minimum inhibitory concentration (MIC) values for itraconazole and amphotericin B. Antifungal susceptibility testing was performed on *Sporothrix brasiliensis* isolates against **A** itraconazole and **B** amphotericin B according to the CLSI M38-A2 microbroth dilution protocol. Isolates were tested in the mycelial phase, and MICs were determined after 72 h of incubation at 30 °C. The number of isolates is displayed above the bar

AFST according to CLSI microbroth dilution was performed for all isolates in the mycelial phase. For itraconazole, a bimodal distribution was observed with 24% (107/450) of isolates classified as NWT (Fig. 2A). At 16 µg/mL, 81 NWT isolates demonstrated partial growth inhibition, estimated at ~ 50% as compared to growth control, while the growth of the other 26 NWT isolates was comparable to growth control. For posaconazole, 20% (89/450) of isolates were NWT (Supplementary Figure S1). MICs for voriconazole, isavuconazole and fluconazole were mostly ≥ 16 µg/mL (Supplementary Figure S1). For amphotericin B, 7% (31/450) of isolates were NWT, with two isolates presenting MICs ≥16 µg/mL (Fig. 2B). Nine isolates were NWT for both itraconazole and amphotericin B.

**Fig. 3:**
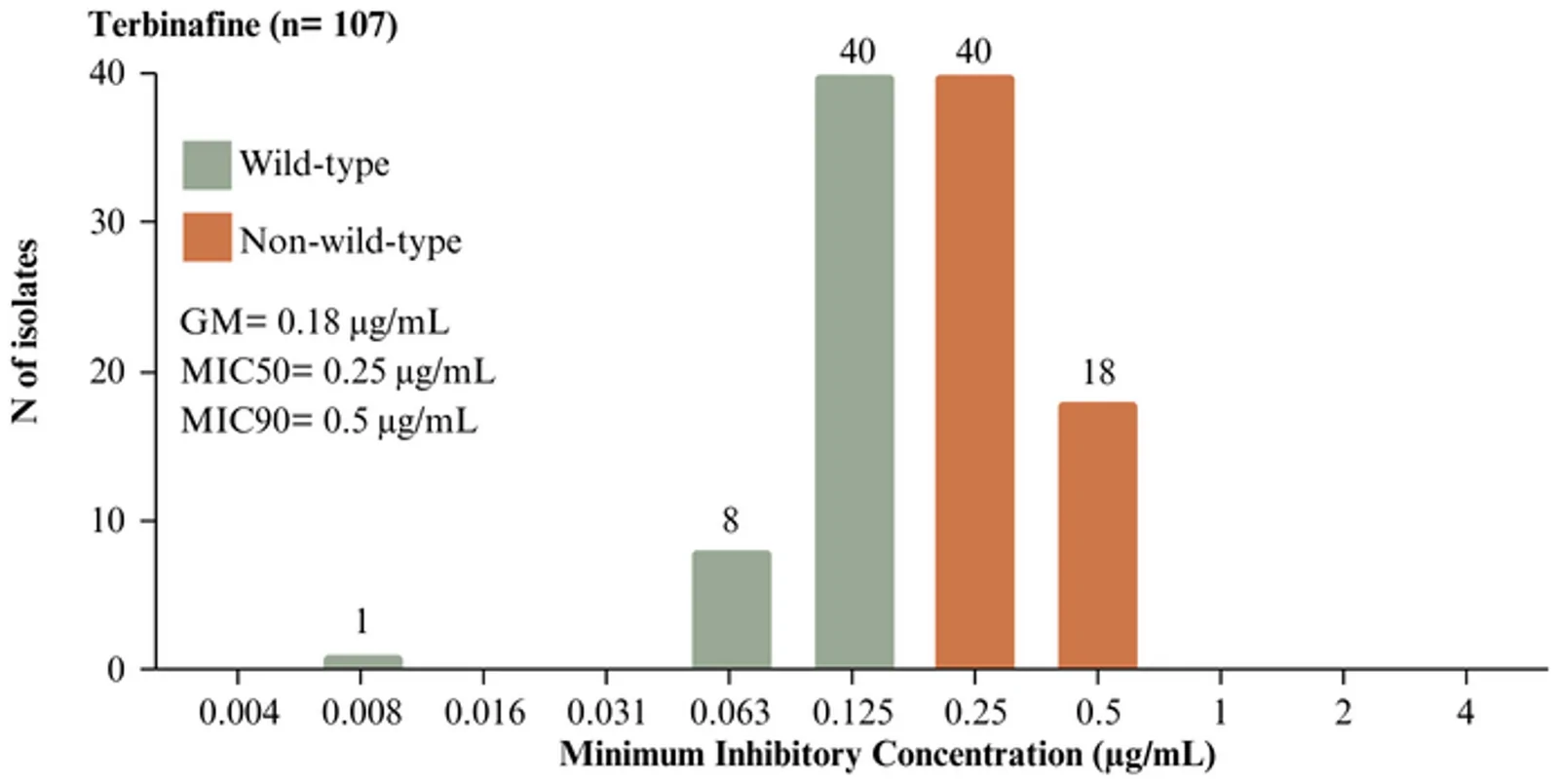
Distribution of minimum inhibitory concentration (MIC) values for terbinafine. Antifungal susceptibility testing was performed on NWT itraconazole *Sporothrix brasiliensis* isolates against terbinafine according to the CLSI M38-A2 microbroth dilution protocol. Isolates were tested in the mycelial phase, and MICs were determined after 72 h of incubation at 30 °C. The number of isolates is displayed above the bar

Next, antifungal susceptibility against terbinafine was determined among the itraconazole NWT isolates. A normal distribution was found with 54% (58/107) being NWT for terbinafine (Fig. 3). Two isolates were NWT for all three antifungals tested. MIC results for individual isolates are provided in Supplementary Material 1.

### Epidemiological Distribution Isolates with Reduced Itraconazole Susceptibility

**Fig. 4:**
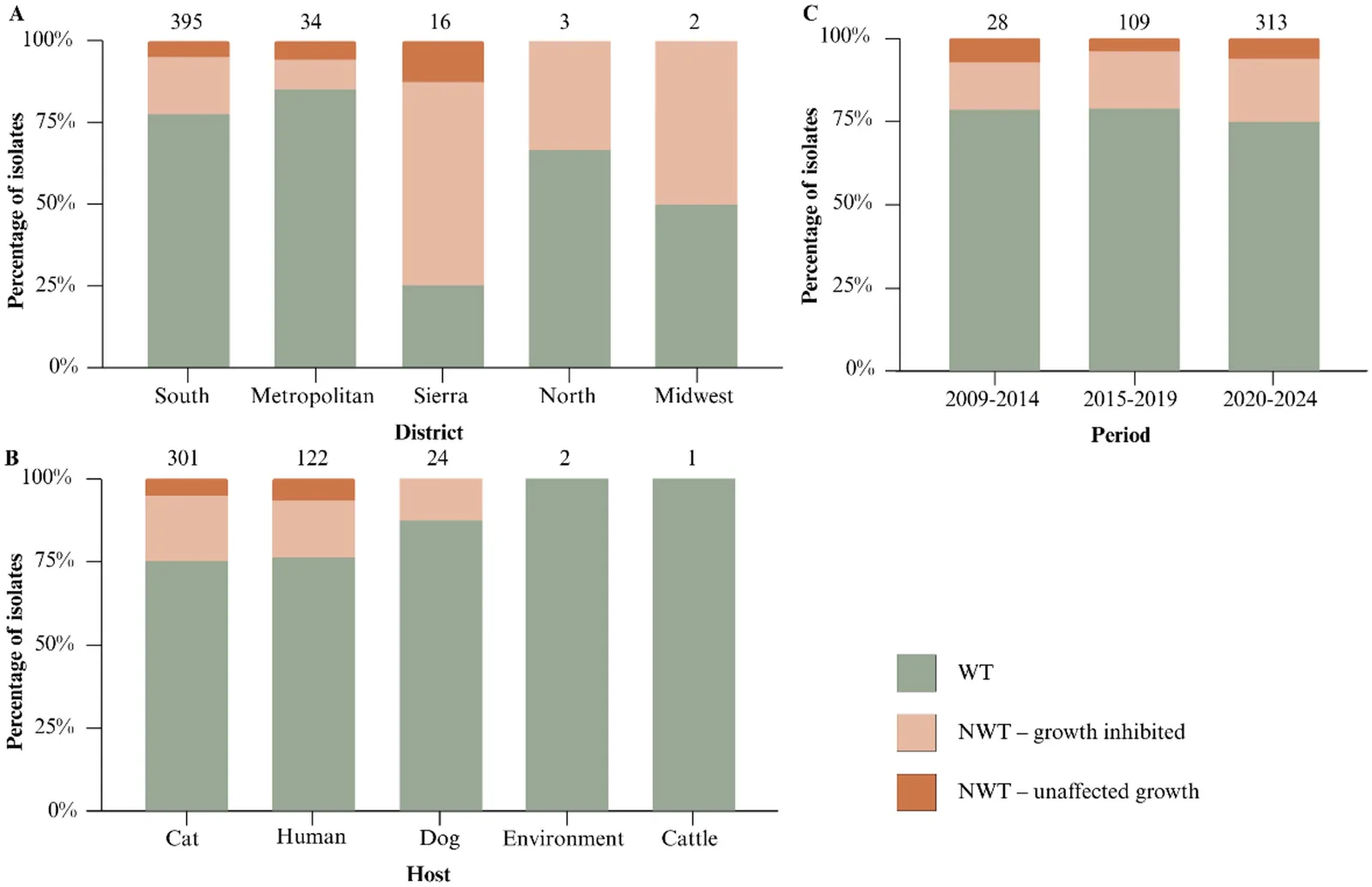
Distribution of WT and NWT isolates per district, host and period. Proportion of itraconazole WT and NWT *Sporothrix brasiliensis* isolates from Rio Grande do Sul by different **A** districts (*p* < 0·05), **B** hosts (*p* > 0·05) and **C** time periods (*p* > 0·05). The number of isolates is displayed above the bar

The distribution of WT and NWT isolates for itraconazole was determined. Non-wild-type were divided in unaffected growth and partial growth inhibition, for the different health districts, time periods, and hosts. In health districts South and Metropolitan, 22.2% (88/395) and 14.7% (5/34) of isolates, respectively, were NWT, while in the Sierra district this percentage was considerably higher (*p*<0.05) (75.0%; 12/16, Fig. 4A). NWT isolates with unaffected growth were found in the Metropolitan district, in the South and Sierra.

When analysed by host, rates of NWT isolates for itraconazole were very similar among cats and humans (24.9% and 23.8%, respectively), while also the rate of NWT isolates with unaffected growth was not different (*p*>0.05) (Fig. 4B). For dogs, 14.3% of isolates showed a reduced itraconazole susceptibility, while none of the NWT isolates showed unaffected growth inhibition. The numbers of isolates from the environment and cattle were too low to compare. Over time, the percentage of NWT isolates and NWT isolates with unaffected growth was comparable (Fig. 4C).

### Short Tandem Repeat Genotyping

**Fig. 5:**
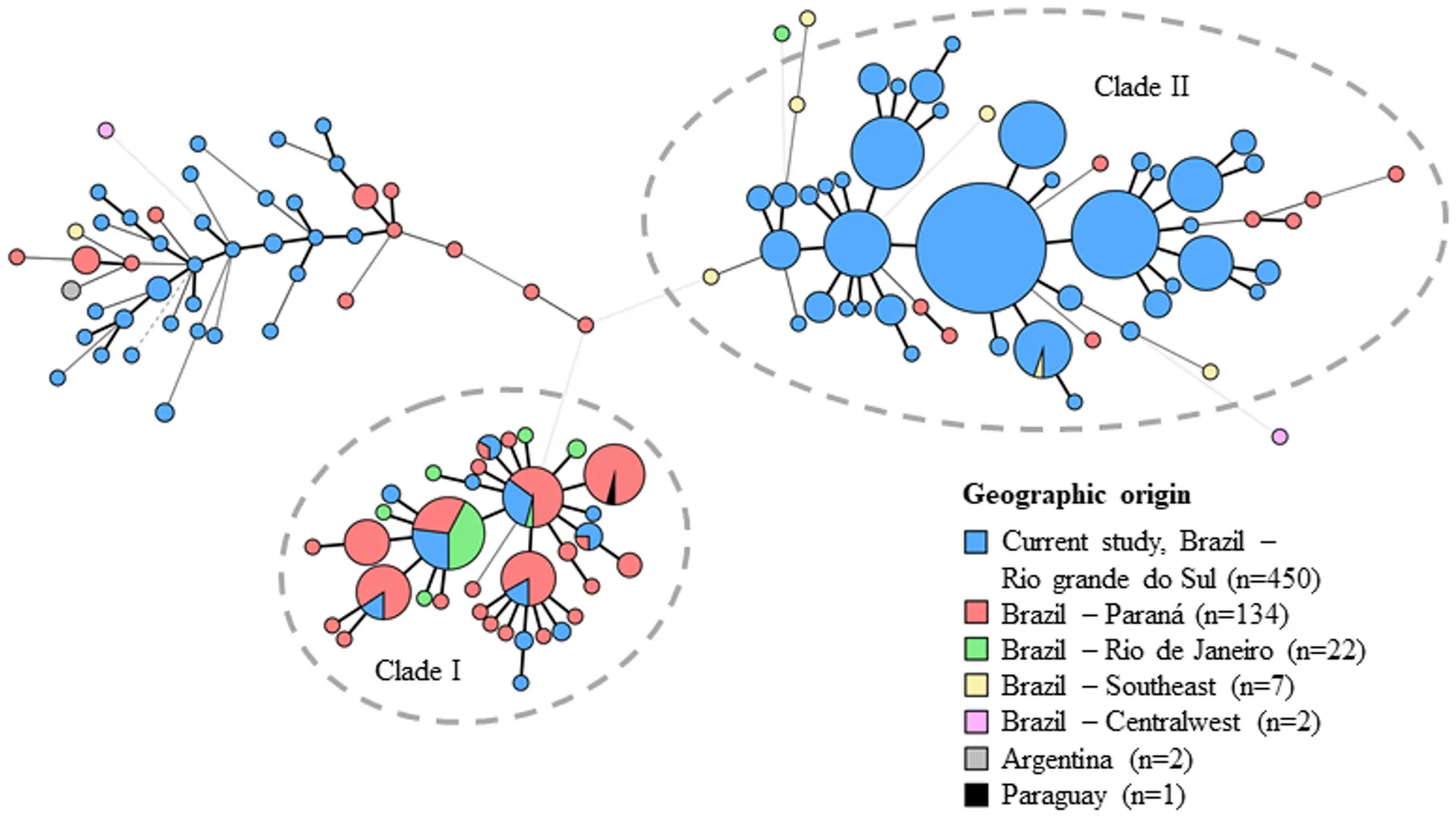
Genetic variation of *Sporothrix brasiliensis* isolates from different regions. Minimum spanning tree of *S. brasiliensis* isolates from Rio Grande do Sul (RS), demonstrating genetic differences between non-RS isolates from previously published studies and all RS isolates from the current study. Branch lengths of the tree indicate similarity between isolates with thick solid lines (variation in one STR marker), thin solid lines (variation in two markers), thin dashed lines (variation in three markers) and thin dotted lines (variation in four or more markers)

STR genotyping was performed on all 450 *S. brasiliensis* isolates, which resulted in a total of 82 genotypes. When compared to previously genotyped isolates, the isolates grouped in two main clades with large clusters and highly related genotypes and a third clade with small clusters of less related genotypes (Fig. 5). The largest clade, with 378 isolates from this study, mainly harboured isolates from the current study (Rio Grande do Sul) and demonstrated clusters up to 107 isolates per cluster. Besides this Rio Grande do Sul associated clade (Clade II), the second main clade, previously identified as the Rio de Janeiro (RJ) clade (Clade I), contained clusters up to 32 isolates per cluster, with at most 9 isolates per cluster from the current study [21]. In total there were 36 isolates from the current study that allocated to Clade I. The remaining 36 isolates from this study grouped with isolates mostly from Paraná state in southern Brazil with 5 isolates per cluster at most (Clade III).

**Fig. 6:**
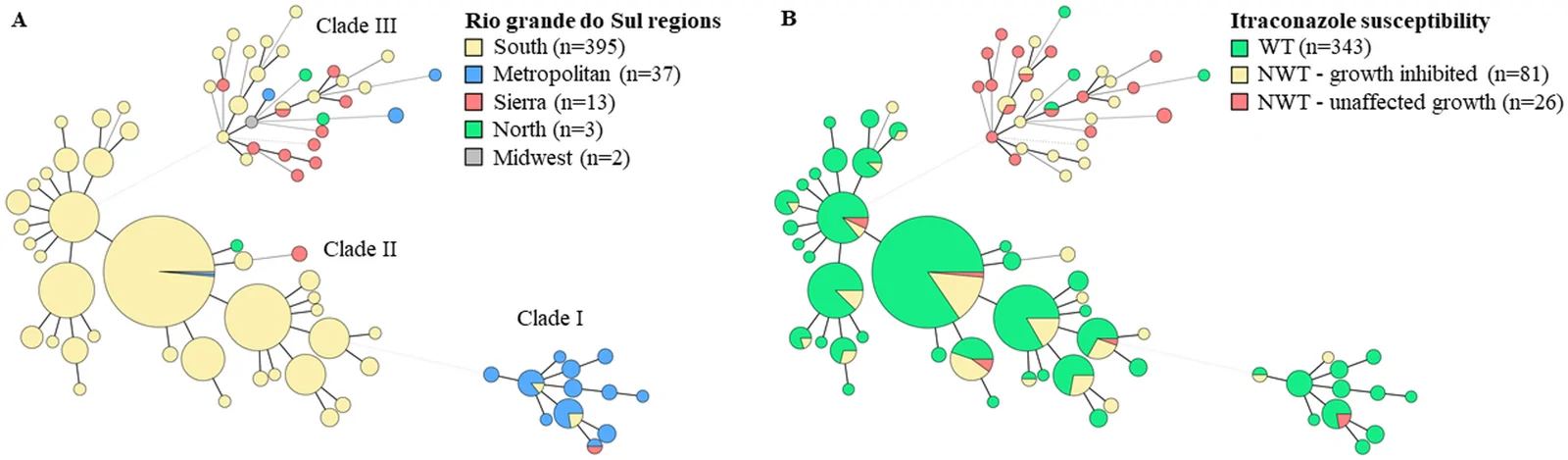
Genetic variation of *Sporothrix brasiliensis* isolates from the current study and their intraconazole in vitro susceptibility profile. Minimum spanning trees of *S. brasiliensis* isolates from Rio Grande do Sul (RS) with, **A** the different Health districts of RS; **B** RS isolates grouped by itraconazole susceptibility profile. Branch lengths of the tree indicate similarity between isolates with thick solid lines (variation in one marker), thin solid lines (variation in two markers), thin dashed lines (variation in three markers) and thin dotted lines (variation in four or more markers)

Analysing the geographic origin of the different isolates from this study only, we found that isolates in Clade II were mostly found in the southern region of the state, while isolates from Clade I were predominantly associated with the Metropolitan district (Fig. 6A). Isolates from the third clade originated from all regions. Furthermore, the genetic variation within Clades I and II was much smaller, with more than 99% of isolates differing by at most one STR marker from their most closely related isolate with a distinct STR profile, whereas only 46% of isolates in Clade III were closely related. When comparing the distribution of clades over time in Rio Grande do Sul, a clear predominance of Clade II was observed throughout all periods, along with isolates from the residual group, which were also present across all time points. In contrast, isolates from Clade I appeared only in the most recent period (Fig. 8).

Next, we determined the genotypic distribution of itraconazole WT vs. NWT isolates. This distribution was highly different between genetic groups as the relative number of NWT isolates in Clade II (72/378, 19%) and I (3/36, 8%) clade was much lower than the 32 out of 36 isolates (89%) observed in Clade III (*p*<0.05) (Fig. 6B, Supplementary Figure S2A). Moreover, this third clade also harboured relatively more NWT isolates for itraconazole with unaffected growth as compared to Clade I and II (Supplementary Figure S2B).

**Fig. 7:**
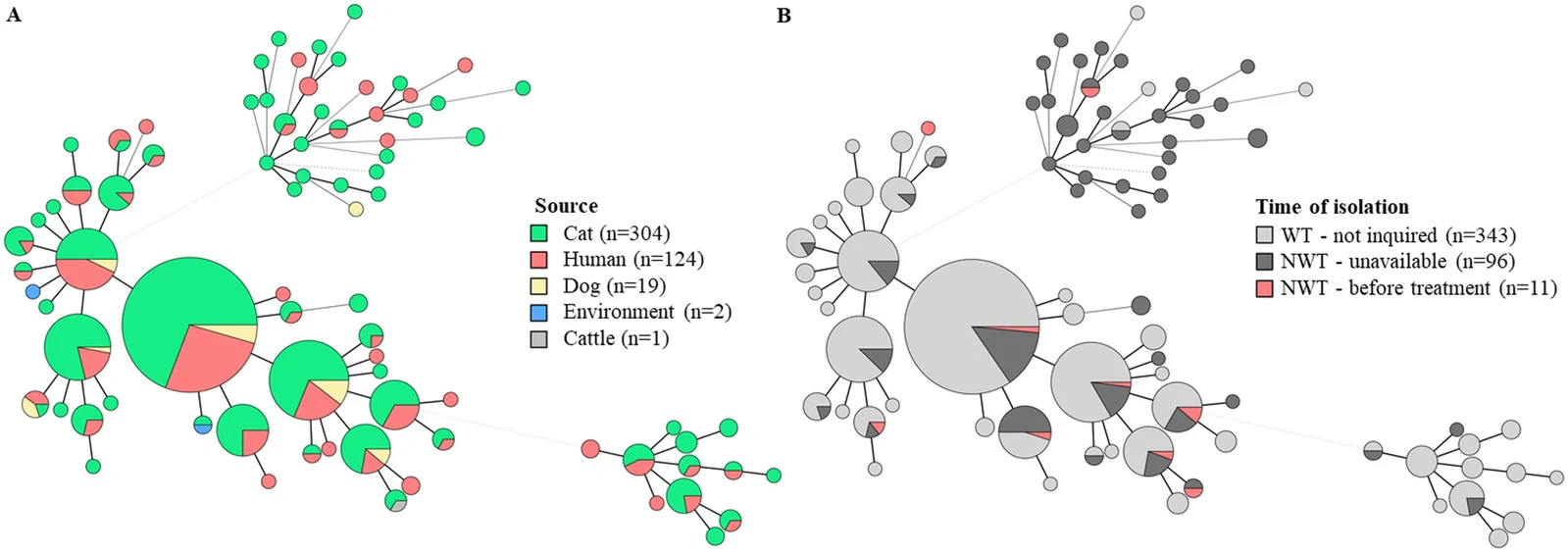
Genetic similarity of *Sporothrix brasiliensis* isolates from different sources and with NWT itraconazole MICs, zoonotically transmitted. Minimum spanning tree of *Sporothrix brasiliensis* isolates from Rio Grande do Sul indicating genetic similarities for **A** different host species; **B** for itraconazole NWT strains, isolated before itraconazole treatment. Onset of treatment in cases with itraconazole-susceptible isolates were not inquired. Branch lengths of the tree indicate similarity between isolates with thick solid lines (variation in one marker), thin solid lines (variation in two markers), thin dashed lines (variation in three markers) and thin dotted lines (variation in four or more markers)

**Fig. 8:**
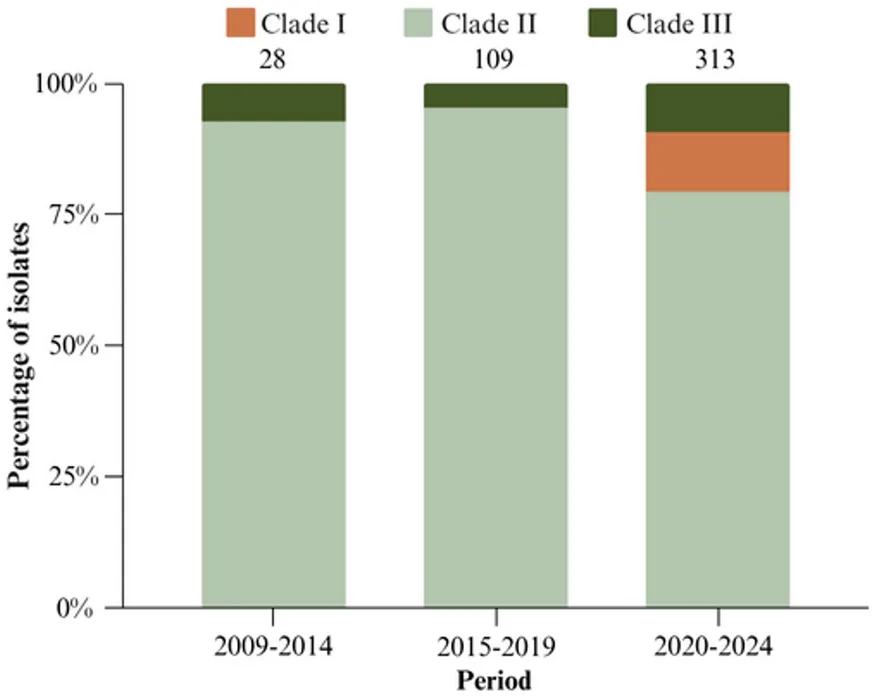
Temporal distribution of *Sporothrix brasiliensis* clades in Rio Grande do Sul state. *Sporothrix brasiliensis* isolates from Rio Grande do Sul (n = 450) were compared according to genetic clade and time of isolation

### Zoonotic Transmission of Strains with Reduced Itraconazole Susceptibility

To investigate whether the isolates with reduced itraconazole susceptibility might be transmitted from cats to humans, we first determined whether identical genotypes were found for the different hosts. As expected, nearly all clusters consisted of both human and feline isolates, indicating widespread zoonotic transmission (Fig. 7A). Then from all isolates NWT for itraconazole, we aimed to collect information about the onset of itraconazole treatment in regard to the isolation of the *S. brasiliensis* strain. All 11 isolates with available information, originating from human patients from the public specialized reference service for diagnosis and treatment of sporotrichosis in southern (University Hospital Miguel Riet Corrêa Jr. from Federal University of Rio Grande) [23], were collected prior to antifungal treatment (Fig. 7B, Supplementary Figure S3).

## Discussion

In this study, the antifungal susceptibility and genetic relatedness of *S. brasiliensis* isolates from Rio Grande do Sul, the southernmost state of Brazil, were analysed. A significant percentage of isolates exhibited high MIC values for itraconazole, the main antifungal agent used to treat sporotrichosis. Moreover, some of these isolates appeared to be collected from humans before the start of itraconazole treatment suggesting cat-to-human transmission of strains with reduced susceptibility. Finally, two novel clades were identified: Clade II with large clusters with isolates mostly all originating from the South region and Clade III with less clusters, higher genetic variability, isolates from different RS regions and high percentage of NWT itraconazole isolates.

Itraconazole AFST demonstrated a bimodal distribution with 24% NWT isolates of *S. brasiliensis,* exhibiting high MIC values (≥ 16 µg/mL). Some of these isolates exhibited no growth reduction at all despite high drug concentrations, whereas others showed a clear partially reduction in growth. These isolates with growth reduction could reflect heteroresistance, in which part of the inoculum was susceptible while other sub-populations were resistant. Another explanation for the different growth pattern may be associated with distinct underlying resistance mechanisms. Some isolates may display tolerance, characterized by reduced but sustained growth, whereas others may harbour more pronounced resistance mechanisms that allow growth even at high antifungal concentrations. Although previous studies have also reported similar high MICs for some *S. brasiliensis* isolates, with one study reporting a bimodal distribution, the number of analysed isolates was relatively small [10, 24, 25]. Moreover, there has been some debate about AFST methodology for *Sporothrix* isolates, with some laboratories not reporting whether the AFST was executed at the pure yeast or filamentous phase. We recently established methods to analyse pure yeast or filamentous phase and included a much larger number of isolates [26]. Thus, we have reinforced and established the presence of reduced itraconazole susceptibility for *S. brasiliensis*. While the impact on clinical outcome in felines and humans remains to be identified, it certainly calls for clinical studies investigating the impact of this reduced itraconazole susceptibility on treatment efficacy.

Besides itraconazole, different other antifungals were tested. Amphotericin B and terbinafine MICs were normally distributed, with a relatively high number of NWT isolates, just one or two dilution factors above the ECV [27]. These findings were likely method-dependent and do not directly suggest an actual reduced susceptibility for these drugs. MICs for voriconazole, isavuconazole and fluconazole were ≥ 16 µg/mL for most isolates. As for itraconazole, a similar bimodal distribution was observed for posaconazole. The main mechanisms of antifungal azole resistance in *Sporothrix* spp. are only partly understood. Recent studies have reported genetic variations in genes involved in resistance pathways, alterations related to aneuploidy and chromosomal variation [10, 28, 29]. Variations in the *cyp51* gene, like the G448S mutation, are most often described, suggesting changes in the target enzyme of itraconazole. While alternative resistance mechanisms are rarely investigated, one study highlighted the contribution of aneuploidy to amphotericin B and itraconazole reduced susceptibility in *S. brasiliensis* [30]. Last, different adaptive responses under antifungal stress might alter the growth pattern of isolates.These genetic and phenotypic variations may help explain the different growth behaviours observed among the resistant isolates.

The epidemiological distribution of isolates with reduced itraconazole susceptibility was investigated. Remarkably, Sierra, a northern region in Rio Grande do Sul, appeared to have more isolates with a NWT itraconazole profile. Subsequent STR genotyping demonstrated that this was due to a different genotype present in this region, a genotype that was also previously found in Paraná state. Thus, regional differences in regard to itraconazole susceptibility were likely due to regional presence of *S. brasiliensis* genotypes. Temporal analysis did not show an increase in the proportion of isolates with high itraconazole MICs over time. While this may be due to the low number of isolates available from earlier years, it is important to emphasize that the absolute number of sporotrichosis cases, and thus also the number of isolates with high itraconazole MICs, has been increasing very quickly over the last years, with yet unknown clinical implications. Additionally, the occurrence of itraconazole NWT isolates spread across diverse genotypes indicates the emergence of multiple populations with reduced itraconazole susceptibility. A reduced susceptibility to antifungals or resistance is acquired via therapy in patients or its presence in the environment, mostly via agriculture. The latter is unlikely as *S. brasiliensis* is not isolated from agriculture environments to date. Instead, the long term treatment in human and cat patients is more likely to cause the reduced susceptibility in multiple genetically distinct populations.

Three major clades were identified, comprising 82 distinct genotypes. While Clade I and II demonstrated many large clusters, indicating clonal transmission, there was a third clade with more genetic variability and small clusters. At this moment it is not evident whether this clade contains isolates that were independently introduced from the environment or were also clonal transmitted but with much lower transmission potential. The larger variation in STR markers in this clade, as compared to Clade I and II, support the former hypothesis, but WGS SNP analysis should be performed to confirm this.

From the beginning of the sporotrichosis endemic, Clade II was dominant in Rio Grande do Sul with > 90% of isolates exhibiting this genotype from 2009 to 2019. Clade II genotypes almost exclusively originate from the southern region of Rio Grande do Sul where the endemic also emerged first [14, 17]. Only from 2020 onwards Clade I was found in Rio Grande do Sul, mostly in the Metropolitan Health District located along the coast, likely via imported cats or via feline movements and transmission [31]. These findings correspond well with the initial reports of the sporotrichosis endemic using low-resolution genotypic analyses, which suggested simultaneous but independent emergences in the states of Rio de Janeiro and Rio Grande do Sul [14, 15, 17, 21]. The high and unique transmission potential of both clade I and II likely plays an important role in current sporotrichosis endemic.

The presence of isolates with NWT itraconazole profiles was relatively low for Clade I and II, with 8% and 19%, respectively, while this was higher for Clade III (89%). This indicates that these clades, with high transmission potential, seem to have a lower potential to reduce their susceptibility to itraconazole. Both characteristics are likely due to genetic differences and future studies should identify the genetic factors involved. Then, all large clusters of Clade II harboured isolates with low and high itraconazole MIC values, indicating the reduced susceptibility was therapy-acquired. Moreover, from all 11 human zoonotic cases with NWT-itraconazole isolates and known clinical information, all strains were isolated prior to itraconazole exposure, suggesting cat-to-human transmission of strains with reduced susceptibility. While this indicates that itraconazole susceptibility reduced during treatment of felines, it is not yet known whether this also occurs in human patients treated with itraconazole.

Study limitations include the high number of isolates from the southern region, while some health districts in Rio Grande do Sul were not included, reflecting the convenience-based sampling strategy adopted by collaborating centers and the limited availability of isolates from these regions, where sporotrichosis has emerged more recently. In addition, terbinafine was only tested in high MIC itraconazole isolates. Furthermore, the inferred genetic relatedness between isolates should be confirmed with whole genome sequencing (WGS). Finally, limited availability of clinical information only allowed to identify zoonotic transmission of strains with reduced itraconazole susceptibility in 11 human patients. Due to the retrospective nature of the study, more clinical outcomes could not be retrieved, refraining us from comparing clinical outcomes to genotypes and MIC data.

This study, based on a large and diverse collection of isolates from Rio Grande do Sul, highlights the emergence of *S. brasiliensis* strains with high itraconazole MIC values, showing a bimodal distribution. The detection of transmissible NWT isolates raises concerns regarding the spread of these strains. Furthermore, the coexistence of multiple clades, either with low or high genetic diversity, reveals complex transmission dynamics that extend beyond clonal expansion. These novel findings underscore the critical role of Rio Grande do Sul state in the current sporotrichosis scenario in Brazil and reinforce the urgent need for improved disease management and control strategies to prevent further dissemination of resistant clades. Additionally, the current findings emphasize the importance of future studies focusing on the clinical impact of reduced itraconazole susceptibility and the underlying mechanisms driving its emergence.

## Supplementary Information

Below is the link to the electronic supplementary material.

Supplementary file1 (DOCX 50 KB)

Supplementary file2 (DOCX 113 KB)

Supplementary file3 (DOCX 51 KB)

Supplementary file4 (PPTX 349 KB)

## Data Availability

All data generated during the current study is included in this article or in the supplementary files.
