# Supplementary material for: Unique Genetic Clades and Frequent Zoonotic Transmission of Sporothrix brasiliensis with Reduced Itraconazole Susceptibility
Source: Mycopathologia. 2026 Sep 15;191(5):80. doi: 10.1007/s11046-026-01101-5 (PMC13578069; doi:10.1007/s11046-026-01101-5)
Supplement: Supplementary file 1 [file 11046_2026_1101_MOESM1_ESM.docx]

**Supplementary 1: Epidemiological characteristics and minimum inhibitory concentrations (MICs) for itraconazole, amphotericin B, and terbinafine of all *Sporothrix brasiliensis* isolates included in this study.**

| Identification | District | Host | Period | MIC ITRA | MIC AMB | MIC TERB |
| --- | --- | --- | --- | --- | --- | --- |
| 10 | South | Human | 2009-2014 | >16* | 2 | 0.25 |
| 89 | South | Cat | 2009-2014 | 1 | 2 |  |
| 399 | South | Cat | 2009-2014 | 0.5 | 2 |  |
| 716 | South | Cat | 2009-2014 | 1 | 2 |  |
| 971 | South | Cat | 2009-2014 | 1 | 2 |  |
| 1023 | South | Cat | 2009-2014 | 0.5 | 1 |  |
| 1078 | South | Cat | 2009-2014 | 0.5 | 2 |  |
| 1079 | South | Human | 2009-2014 | 2 | 4 |  |
| 1118 | South | Cat | 2009-2014 | 1 | 4 |  |
| 1464 | South | Cat | 2009-2014 | 1 | 4 |  |
| 1543 | South | Cat | 2009-2014 | 2 | 4 |  |
| 1573 | South | Cat | 2009-2014 | 2 | 4 |  |
| 1644 | South | Cat | 2009-2014 | >16* | 2 | 0.063 |
| 1651 | South | Cat | 2009-2014 | 0.5 | 2 |  |
| 1668 | South | Cat | 2009-2014 | 2 | 2 |  |
| 1705 | South | Cat | 2009-2014 | 2 | 2 |  |
| 1740 | South | Cat | 2009-2014 | 2 | 2 |  |
| 1765 | South | Cat | 2009-2014 | 2 | 2 |  |
| 1801 | South | Cat | 2009-2014 | 1 | 2 |  |
| 1812 | South | Cat | 2009-2014 | 1 | 2 |  |
| 1817 | South | Human | 2009-2014 | >16* | 2 | 0.5 |
| 1878 | South | Human | 2009-2014 | >16 | 1 | 0.125 |
| 1893 | South | Cat | 2009-2014 | 1 | 2 |  |
| 1896 | South | Cat | 2009-2014 | 2 | 2 |  |
| 1936 | South | Human | 2009-2014 | 1 | 2 |  |
| 1940 | South | Human | 2009-2014 | 0.5 | 4 |  |
| 1992 | South | Cat | 2009-2014 | >16* | 2 | 0.063 |
| 2373 | South | Cat | 2009-2014 | >16 | 2 | 0.25 |
| 2615 | South | Cat | 2015-2019 | >16 | 2 | 0.5 |
| 2625 | South | Human | 2015-2019 | 2 | 2 |  |
| 2687 | South | Cat | 2015-2019 | 2 | 4 |  |
| 2691 | South | Environment | 2015-2019 | 2 | 2 |  |
| 2792 | South | Cat | 2015-2019 | 2 | 2 |  |
| 2811 | South | Dog | 2015-2019 | 0.5 | 2 |  |
| 2812 | South | Dog | 2015-2019 | 1 | 2 |  |
| 2898 | South | Cat | 2015-2019 | 2 | 4 |  |
| 3022 | South | Cat | 2015-2019 | 2 | 2 |  |
| 3105 | South | Cat | 2015-2019 | >16 | 4 | 0.125 |
| 3160 | South | Cat | 2015-2019 | 0.5 | 2 |  |
| 3207 | South | Cat | 2015-2019 | 2 | 4 |  |
| 3253 | South | Cat | 2015-2019 | 1 | 2 |  |
| 3354 | South | Cat | 2015-2019 | 1 | 4 |  |
| 3356 | South | Human | 2015-2019 | 2 | 2 |  |
| 3357 | South | Human | 2015-2019 | 1 | 2 |  |
| 3368 | South | Cat | 2015-2019 | 0.5 | 4 |  |
| 3397 | South | Cat | 2015-2019 | 0.5 | 2 |  |
| 3746 | South | Cat | 2015-2019 | 1 | 2 |  |
| 3792 | South | Human | 2015-2019 | 1 | 2 |  |
| 3804 | South | Human | 2015-2019 | 0.5 | 2 |  |
| 3943 | South | Cat | 2015-2019 | 2 | 2 |  |
| 3944 | South | Cat | 2015-2019 | 1 | 2 |  |
| 3952 | South | Human | 2015-2019 | >16 | 2 | 0.25 |
| 4015 | South | Dog | 2015-2019 | 1 | 2 |  |
| 4023 | South | Cat | 2015-2019 | >16* | 2 | 0.125 |
| 4037 | South | Human | 2015-2019 | 0.5 | 4 |  |
| 4044 | South | Cat | 2015-2019 | >16* | 2 | 0.25 |
| 4082 | South | Cat | 2015-2019 | 0.5 | 2 |  |
| 4087 | South | Cat | 2015-2019 | >16 | 2 | 0.5 |
| 4099 | South | Human | 2015-2019 | >16* | 2 | 0.25 |
| 4137 | South | Cat | 2015-2019 | 2 | 2 |  |
| 4163 | South | Cat | 2015-2019 | 2 | 2 |  |
| 4176 | South | Cat | 2015-2019 | 1 | 2 |  |
| 4221 | South | Cat | 2015-2019 | >16* | 4 | 0.5 |
| 4238 | South | Cat | 2015-2019 | >16* | 4 | 0.125 |
| 4253 | South | Human | 2015-2019 | 1 | 2 |  |
| 4306 | South | Cat | 2015-2019 | 2 | 2 |  |
| 4314 | South | Cat | 2015-2019 | >16* | 8 | 0.5 |
| 4335 | South | Cat | 2015-2019 | >16* | 2 | 0.25 |
| 4349 | South | Human | 2015-2019 | 0.5 | 2 |  |
| 4419 | South | Cat | 2015-2019 | >16* | 2 | 0.25 |
| 4441 | South | Human | 2015-2019 | 1 | 2 |  |
| 4466 | South | Dog | 2015-2019 | 1 | 4 |  |
| 4470 | South | Human | 2015-2019 | 0.5 | 4 |  |
| 4475 | South | Cat | 2015-2019 | 2 | 4 |  |
| 4519 | South | Human | 2015-2019 | 2 | 2 |  |
| 4559 | South | Cat | 2015-2019 | >16* | 2 | 0.5 |
| 4564 | South | Cat | 2015-2019 | 1 | 2 |  |
| 4569 | South | Cat | 2015-2019 | 2 | 2 |  |
| 4574 | South | Cat | 2015-2019 | 1 | 4 |  |
| 4666 | South | Cat | 2015-2019 | >16* | 2 | 0.063 |
| 4674 | South | Human | 2015-2019 | 0.5 | 2 |  |
| 4692 | South | Cat | 2015-2019 | 2 | 2 |  |
| 4700 | South | Human | 2015-2019 | 1 | 4 |  |
| 4720 | South | Cat | 2015-2019 | 1 | 4 |  |
| 4725 | South | Cat | 2015-2019 | >16* | 2 | 0.125 |
| 4748 | South | Cat | 2015-2019 | 1 | 2 |  |
| 4770 | South | Dog | 2015-2019 | 1 | 2 |  |
| 4816 | South | Cat | 2015-2019 | 2 | 2 |  |
| 4819 | South | Cat | 2015-2019 | 2 | 2 |  |
| 4825 | South | Cat | 2015-2019 | 1 | 2 |  |
| 4860 | South | Human | 2015-2019 | 1 | 2 |  |
| 4879 | South | Dog | 2015-2019 | 2 | 2 |  |
| 4882 | South | Cat | 2015-2019 | 2 | 2 |  |
| 4906 | South | Human | 2015-2019 | 2 | 2 |  |
| 4910 | South | Human | 2015-2019 | >16* | 2 | 0.5 |
| 4924 | South | Cat | 2015-2019 | 2 | 2 |  |
| 4931 | South | Cat | 2015-2019 | 1 | 4 |  |
| 4941 | South | Dog | 2015-2019 | 2 | 2 |  |
| 4948 | South | Cat | 2015-2019 | 0.5 | 4 |  |
| 4986 | South | Cat | 2015-2019 | 2 | 2 |  |
| 4996 | South | Cat | 2015-2019 | 2 | 2 |  |
| 5010 | South | Cat | 2015-2019 | 0.5 | 4 |  |
| 5034 | South | Human | 2015-2019 | 0.5 | 4 |  |
| 5053 | South | Cat | 2015-2019 | 2 | 4 |  |
| 5064 | South | Human | 2015-2019 | 2 | 2 |  |
| 5080 | South | Human | 2015-2019 | 1 | 2 |  |
| 5117 | South | Dog | 2015-2019 | 2 | 2 |  |
| 5125 | South | Human | 2015-2019 | 2 | 2 |  |
| 5127 | South | Human | 2015-2019 | 0.5 | 4 |  |
| 5137 | South | Cat | 2015-2019 | 1 | 2 |  |
| 5150 | South | Human | 2015-2019 | 1 | 2 |  |
| 5182 | South | Human | 2015-2019 | 2 | 4 |  |
| 5183 | South | Cat | 2015-2019 | 1 | 4 |  |
| 5197 | South | Human | 2015-2019 | 1 | 4 |  |
| 5211 | South | Cat | 2015-2019 | 1 | 2 |  |
| 5243 | South | Cat | 2015-2019 | 1 | 2 |  |
| 5261 | South | Cat | 2015-2019 | 2 | 4 |  |
| 5318 | South | Cat | 2015-2019 | 1 | 2 |  |
| 5328 | South | Cat | 2015-2019 | >16* | 2 | 0.25 |
| 5361 | South | Human | 2015-2019 | 1 | 8 |  |
| 5385 | South | Dog | 2015-2019 | 1 | 2 |  |
| 5416 | South | Cat | 2015-2019 | 2 | 4 |  |
| 5422 | South | Cat | 2015-2019 | >16* | 2 | 0.25 |
| 5425 | South | Human | 2015-2019 | 1 | 2 |  |
| 5477 | South | Cat | 2015-2019 | 0.5 | 2 |  |
| 5489 | South | Cat | 2015-2019 | 2 | 2 |  |
| 5547 | South | Cat | 2015-2019 | >16* | 2 | 0.125 |
| 5562 | South | Human | 2015-2019 | 0.5 | 2 |  |
| 5573 | South | Dog | 2015-2019 | 2 | 4 |  |
| 5584 | South | Cat | 2015-2019 | >16* | 8 | 0.125 |
| 5585 | South | Human | 2015-2019 | 0.5 | 2 |  |
| 5608 | South | Cat | 2015-2019 | 0.5 | 2 |  |
| 5657 | South | Cat | 2015-2019 | 2 | 2 |  |
| 5659 | South | Human | 2015-2019 | 4 | 2 |  |
| 5698 | South | Cat | 2020-2024 | 2 | 1 |  |
| 5723 | South | Cat | 2020-2024 | 1 | 4 |  |
| 5762 | South | Cat | 2020-2024 | >16 | 2 | 0.125 |
| 5783 | South | Cat | 2020-2024 | 4 | 2 |  |
| 5806 | South | Human | 2020-2024 | 1 | 2 |  |
| 5822 | South | Cat | 2020-2024 | >16* | 2 | 0.25 |
| 5829 | South | Cat | 2020-2024 | 1 | 4 |  |
| 5845 | South | Cat | 2020-2024 | 16* | 4 | 0.063 |
| 5872 | South | Human | 2020-2024 | 0.25 | 2 |  |
| 5885 | South | Cat | 2020-2024 | 1 | 4 |  |
| 5913 | South | Cat | 2020-2024 | 2 | 2 |  |
| 5953 | South | Cat | 2020-2024 | 2 | 2 |  |
| 5957 | South | Cat | 2020-2024 | 2 | 2 |  |
| 6012 | South | Cat | 2020-2024 | 0.5 | 2 |  |
| 6094 | South | Cat | 2020-2024 | >16 | 4 | 0.25 |
| 6181 | South | Human | 2020-2024 | 1 | 2 |  |
| 6280 | South | Cat | 2020-2024 | 2 | 2 |  |
| 6320 | South | Cat | 2020-2024 | 1 | 2 |  |
| 6332 | South | Cat | 2020-2024 | 2 | 2 |  |
| 6349 | South | Cat | 2020-2024 | 0.5 | 2 |  |
| 6375 | South | Cat | 2020-2024 | 1 | 4 |  |
| 6415 | Sierra | Cat | 2020-2024 | 2 | 4 |  |
| 6441 | South | Human | 2020-2024 | 1 | 4 |  |
| 6491 | South | Cat | 2020-2024 | 1 | 4 |  |
| 6556 | South | Cat | 2020-2024 | 1 | 2 |  |
| 6565 | South | Cat | 2020-2024 | 2 | 4 |  |
| 6613 | South | Dog | 2020-2024 | >16* | 2 | 0.125 |
| 6614 | South | Dog | 2020-2024 | 1 | 1 |  |
| 6633 | South | Cat | 2020-2024 | >16* | 4 | 0.125 |
| 6634 | South | Cat | 2020-2024 | 0.5 | 2 |  |
| 6677 | South | Cat | 2020-2024 | 2 | 8 |  |
| 6693 | South | Cat | 2020-2024 | 2 | 2 |  |
| 6735 | South | Cat | 2020-2024 | 2 | 4 |  |
| 6746 | South | Cat | 2020-2024 | 2 | 2 |  |
| 6753 | South | Cat | 2020-2024 | >16* | 2 | 0.25 |
| 6809 | South | Cat | 2020-2024 | 2 | 2 |  |
| 6810 | South | Cat | 2020-2024 | 4 | 2 |  |
| 6885 | South | Cat | 2020-2024 | 2 | 2 |  |
| 6898 | South | Human | 2020-2024 | 0.5 | 4 |  |
| 6927 | South | Cat | 2020-2024 | >16 | 2 | 0.25 |
| 6951 | South | Human | 2020-2024 | 2 | 2 |  |
| 7027 | South | Cat | 2020-2024 | 2 | 4 |  |
| 7048 | South | Cat | 2020-2024 | 4 | 2 |  |
| 7082 | South | Cat | 2020-2024 | >16* | 4 | 0.125 |
| 7132 | South | Cat | 2020-2024 | 2 | 2 |  |
| 7202 | South | Cat | 2020-2024 | >16* | 4 | 0.125 |
| 7230 | South | Cat | 2020-2024 | 2 | 2 |  |
| 7255 | South | Cat | 2020-2024 | 2 | 2 |  |
| 7277 | South | Cat | 2020-2024 | 1 | 2 |  |
| 7341 | South | Cat | 2020-2024 | 0.5 | 4 |  |
| 7346 | South | Cat | 2020-2024 | 2 | 2 |  |
| 7451 | Metropolitan | Human | 2020-2024 | 1 | 4 |  |
| 7452 | Metropolitan | Human | 2020-2024 | 0.5 | 2 |  |
| 7457 | Metropolitan | Human | 2020-2024 | 2 | >16* |  |
| 7458 | Metropolitan | Human | 2020-2024 | 1 | 4 |  |
| 7545 | South | Cat | 2020-2024 | 2 | 2 |  |
| 7550 | South | Cat | 2020-2024 | >16* | 2 | 0.25 |
| 7559 | South | Cat | 2020-2024 | 0.5 | 2 |  |
| 7604 | South | Dog | 2020-2024 | 2 | 8 |  |
| 7641 | South | Human | 2020-2024 | 2 | 2 |  |
| 7648 | South | Human | 2020-2024 | 2 | 2 |  |
| 7675 | South | Cat | 2020-2024 | >16* | 2 | 0.125 |
| 7683 | South | Dog | 2020-2024 | 1 | 2 |  |
| 7711 | South | Cat | 2020-2024 | >16* | 2 | 0.5 |
| 7736 | South | Human | 2020-2024 | 0.5 | 2 |  |
| 7762 | South | Cat | 2020-2024 | 4 | 8 |  |
| 7810 | South | Cat | 2020-2024 | 0.5 | 4 |  |
| 7874 | South | Human | 2020-2024 | 2 | 4 |  |
| 7875 | South | Cat | 2020-2024 | 0.5 | 2 |  |
| 7885 | South | Cat | 2020-2024 | 1 | 2 |  |
| 7899 | South | Cat | 2020-2024 | 1 | 2 |  |
| 7970 | South | Cat | 2020-2024 | 2 | 2 |  |
| 8079 | South | Human | 2020-2024 | >16* | 1 | 0.125 |
| 8155 | South | Cat | 2020-2024 | >16* | 2 | 0.125 |
| 8156 | South | Cat | 2020-2024 | >16* | 4 | 0.25 |
| 8159 | South | Cat | 2020-2024 | 2 | 2 | 0.125 |
| 8214 | South | Dog | 2020-2024 | 2 | 2 |  |
| 8216 | South | Human | 2020-2024 | 1 | 2 |  |
| 8234 | South | Cat | 2020-2024 | 2 | 2 |  |
| 8446 | South | Cat | 2020-2024 | 2 | 4 |  |
| 8622 | South | Human | 2020-2024 | 1 | 2 |  |
| 8694 | South | Cat | 2020-2024 | 0.5 | 2 |  |
| 8764 | South | Human | 2020-2024 | 1 | 2 |  |
| 8782 | South | Cat | 2020-2024 | 2 | 4 |  |
| 8932 | South | Cat | 2020-2024 | >16* | 8 | 0.063 |
| 9032 | South | Cat | 2020-2024 | 2 | 2 |  |
| 9041 | South | Cat | 2020-2024 | 2 | 2 |  |
| 9044 | South | Human | 2020-2024 | >16* | 2 | 0.125 |
| 9048 | South | Human | 2020-2024 | 4 | 2 |  |
| 9164 | South | Human | 2020-2024 | 4 | 4 |  |
| 9209 | South | Human | 2020-2024 | >16* | 1 | 0.25 |
| 9305 | South | Cat | 2020-2024 | >16* | 2 | 0.125 |
| 9328 | South | Cat | 2020-2024 | 2 | 2 |  |
| 9416 | South | Cat | 2020-2024 | 1 | 4 |  |
| 9571 | South | Cat | 2020-2024 | >16* | 2 | 0.125 |
| 9594 | South | Cat | 2020-2024 | 1 | 2 |  |
| 9604 | South | Human | 2020-2024 | 1 | 2 |  |
| 9823 | South | Human | 2020-2024 | 0.5 | 2 |  |
| 9824 | South | Human | 2020-2024 | 0.5 | 1 |  |
| 9868 | South | Cat | 2020-2024 | 1 | 2 |  |
| 10010 | South | Environment | 2020-2024 | 2 | 1 |  |
| 10024 | South | Cat | 2020-2024 | 2 | 2 |  |
| 10068 | South | Cat | 2020-2024 | 1 | 2 |  |
| 10091 | South | Human | 2020-2024 | 2 | 2 |  |
| 10100 | South | Cat | 2020-2024 | 2 | 2 |  |
| 10104 | South | Human | 2020-2024 | 2 | 2 |  |
| 10109 | South | Human | 2020-2024 | >16* | 1 | 0.063 |
| 10110 | South | Human | 2020-2024 | 1 | 4 |  |
| 10119 | North | Human | 2020-2024 | 4 | 8 |  |
| 10133 | South | Dog | 2020-2024 | 1 | 2 |  |
| 10155 | South | Human | 2020-2024 | 2 | 2 |  |
| 10178 | South | Cat | 2020-2024 | 1 | 2 |  |
| 10182 | South | Cat | 2020-2024 | 1 | 2 |  |
| 10195 | South | Cat | 2020-2024 | 0.5 | 4 |  |
| 10225 | South | Cat | 2020-2024 | 1 | 2 |  |
| 10331 | South | Human | 2020-2024 | 1 | 2 |  |
| 10336 | South | Cat | 2020-2024 | 2 | 2 |  |
| 10486 | South | Cat | 2020-2024 | 2 | 2 |  |
| 10487 | South | Cat | 2020-2024 | 2 | 2 |  |
| 10540 | Sierra | Cat | 2015-2019 | >16* | 1 | 0.125 |
| 10541 | Sierra | Cat | 2020-2024 | >16 | 2 | 0.125 |
| 10542 | Sierra | Cat | 2020-2024 | >16* | 1 | 0.125 |
| 10543 | Sierra | Cat | 2020-2024 | >16* | 1 | 0.25 |
| 10544 | Sierra | Cat | 2020-2024 | 2 | 2 |  |
| 10545 | Sierra | Cat | 2020-2024 | >16* | 2 | 0.125 |
| 10546 | Sierra | Dog | 2020-2024 | 4 | 2 |  |
| 10547 | Sierra | Cat | 2020-2024 | >16* | 2 | 0.125 |
| 10548 | Sierra | Cat | 2020-2024 | 16* | 1 | 0.25 |
| 10549 | Sierra | Cat | 2020-2024 | >16* | 1 | 0.25 |
| 10550 | Sierra | Cat | 2020-2024 | >16* | 2 | 0.25 |
| 10551 | Sierra | Cat | 2020-2024 | >16* | 1 | 0.25 |
| 10798 | South | Cat | 2020-2024 | 2 | 4 |  |
| 10800 | South | Cat | 2020-2024 | 2 | 4 |  |
| 10801 | South | Human | 2020-2024 | >16* | 16 | 0.5 |
| 10823 | Sierra | Cat | 2020-2024 | 2 | 4 |  |
| 10824 | Sierra | Cat | 2020-2024 | >16 | 1 | 0.25 |
| 10825 | Sierra | Dog | 2020-2024 | >16* | 2 | 0.25 |
| 10829 | South | Human | 2020-2024 | 1 | 4 |  |
| 10831 | South | Human | 2020-2024 | 1 | 2 |  |
| 10864 | South | Cat | 2020-2024 | 2 | 2 |  |
| 10865 | South | Cat | 2020-2024 | 1 | 2 |  |
| 10886 | South | Cat | 2020-2024 | 2 | 2 |  |
| 10888 | South | Cat | 2020-2024 | 2 | 2 |  |
| 10901 | South | Human | 2020-2024 | 2 | 2 |  |
| 10903 | South | Human | 2020-2024 | >16 | 2 | 0.5 |
| 10918 | South | Human | 2020-2024 | 0.5 | 2 |  |
| 10949 | South | Cat | 2020-2024 | >16* | 2 | 0.25 |
| 11005 | South | Human | 2020-2024 | 4 | 2 |  |
| 11050 | South | Cat | 2020-2024 | >16* | 2 | 0.125 |
| 11061 | South | Cat | 2020-2024 | 0.5 | 2 |  |
| 11098 | South | Dog | 2020-2024 | 2 | 2 |  |
| 11163 | South | Cat | 2020-2024 | 0.5 | 2 |  |
| 11166 | South | Human | 2020-2024 | 2 | 4 |  |
| 11178 | South | Human | 2020-2024 | >16* | 2 | 0.25 |
| 11200 | South | Cat | 2020-2024 | >16* | 2 | 0.5 |
| 11217 | South | Cat | 2020-2024 | 2 | 4 |  |
| 11224 | South | Cat | 2020-2024 | 1 | 4 |  |
| 11245 | South | Cat | 2020-2024 | >16* | 2 | 0.125 |
| 11256 | South | Cat | 2020-2024 | 2 | 4 |  |
| 11309 | South | Human | 2020-2024 | >16 | 4 | 0.25 |
| 11327 | South | Cat | 2020-2024 | 2 | 4 |  |
| 11330 | South | Cat | 2020-2024 | >16* | 2 | 0.5 |
| 11345 | South | Cat | 2020-2024 | 2 | 2 |  |
| 11346 | South | Human | 2020-2024 | >16* | 2 | 0.25 |
| 11387 | South | Dog | 2020-2024 | 2 | 2 |  |
| 11388 | South | Cat | 2020-2024 | 2 | 4 |  |
| 11392 | South | Cat | 2020-2024 | 4 | 4 |  |
| 11393 | South | Human | 2020-2024 | 2 | 2 |  |
| 11406 | South | Cat | 2020-2024 | 2 | 4 |  |
| 11412 | South | Cat | 2020-2024 | >16 | 1 | 0.5 |
| 11451 | South | Human | 2020-2024 | 2 | 2 |  |
| 11452 | South | Human | 2020-2024 | >16 | 2 | 0.125 |
| 11453 | South | Human | 2020-2024 | >16* | 2 | 0.125 |
| 11454 | South | Cat | 2020-2024 | 2 | 2 |  |
| 11463 | South | Cat | 2020-2024 | 1 | 2 |  |
| 11466 | South | Human | 2020-2024 | 2 | 2 |  |
| 11468 | South | Human | 2020-2024 | 2 | 4 |  |
| 11513 | South | Human | 2020-2024 | 0.5 | 2 |  |
| 11514 | South | Human | 2020-2024 | 0.5 | 2 |  |
| 11515 | South | Human | 2020-2024 | >16* | 4 | 0.125 |
| 11517 | South | Human | 2020-2024 | >16* | 2 | 0.25 |
| 11518 | South | Human | 2020-2024 | >16* | 2 | 0.25 |
| 11523 | South | Cat | 2020-2024 | 2 | 2 |  |
| 11529 | South | Cat | 2020-2024 | 4 | 8 |  |
| 11540 | South | Human | 2020-2024 | >16* | 2 | 0.125 |
| 11581 | South | Cat | 2020-2024 | 1 | 8 |  |
| 11622 | South | Cat | 2020-2024 | 2 | 2 |  |
| 11648 | South | Cat | 2020-2024 | 1 | 2 |  |
| 11675 | South | Cat | 2020-2024 | >16* | 2 | 0.25 |
| 11681 | South | Cat | 2020-2024 | >16* | 2 | 0.25 |
| 11697 | South | Cat | 2020-2024 | 2 | 2 |  |
| 11721 | South | Cat | 2020-2024 | 1 | 2 |  |
| 11726 | South | Cat | 2020-2024 | 1 | 2 |  |
| 11727 | South | Cat | 2020-2024 | >16 | 2 | 0.25 |
| 11728 | South | Cat | 2020-2024 | 2 | 2 |  |
| 11729 | South | Cat | 2020-2024 | 2 | 2 |  |
| 11730 | South | Cat | 2020-2024 | 2 | 2 |  |
| 11732 | South | Cat | 2020-2024 | 2 | 4 |  |
| 11733 | South | Cat | 2020-2024 | 2 | 2 |  |
| 11734 | South | Cat | 2020-2024 | 2 | 2 |  |
| 11736 | South | Cattle | 2020-2024 | 2 | 2 |  |
| 11738 | South | Cat | 2020-2024 | 2 | 2 |  |
| 11740 | South | Cat | 2020-2024 | >16* | 2 | 0.125 |
| 11741 | South | Cat | 2020-2024 | 2 | 2 |  |
| 11743 | South | Cat | 2020-2024 | 1 | 1 |  |
| 11745 | South | Cat | 2020-2024 | 2 | 2 |  |
| 11746 | South | Cat | 2020-2024 | 2 | 8 |  |
| 11747 | South | Cat | 2020-2024 | 2 | 2 |  |
| 11750 | South | Cat | 2020-2024 | 2 | 2 |  |
| 11751 | Metropolitan | Cat | 2020-2024 | 2 | 4 |  |
| 11752 | South | Cat | 2020-2024 | 2 | 4 |  |
| 11755 | Metropolitan | Cat | 2020-2024 | 1 | 8 |  |
| 11756 | Metropolitan | Cat | 2020-2024 | 2 | 2 |  |
| 11758 | Metropolitan | Cat | 2020-2024 | 2 | 4 |  |
| 11768 | South | Human | 2020-2024 | 2 | 2 |  |
| 11781 | South | Dog | 2020-2024 | 2 | 4 |  |
| 11782 | South | Cat | 2020-2024 | 1 | 2 |  |
| 11784 | South | Cat | 2020-2024 | 1 | 2 |  |
| 11792 | South | Cat | 2020-2024 | 2 | 4 |  |
| 11799 | South | Human | 2020-2024 | >16* | 2 | 0.125 |
| 11801 | South | Human | 2020-2024 | 0.5 | 2 |  |
| 11803 | South | Human | 2020-2024 | 1 | 4 |  |
| 11804 | South | Human | 2020-2024 | >16 | 2 | 0.25 |
| 11813 | South | Cat | 2020-2024 | 2 | 8 |  |
| 11839 | South | Human | 2020-2024 | 2 | 2 |  |
| 11846 | South | Human | 2020-2024 | 2 | 2 |  |
| 11847 | South | Cat | 2020-2024 | 2 | 2 |  |
| 11851 | South | Human | 2020-2024 | 2 | 2 |  |
| 11852 | Midwest | Cat | 2020-2024 | 2 | 8 |  |
| 11854 | Midwest | Cat | 2020-2024 | >16* | 2 | 0.5 |
| 11900 | South | Cat | 2020-2024 | 2 | 2 |  |
| 11901 | South | Human | 2020-2024 | 2 | 2 |  |
| 11909 | South | Cat | 2020-2024 | 2 | 4 |  |
| 11911 | South | Cat | 2020-2024 | 2 | 4 |  |
| 11939 | South | Human | 2020-2024 | 2 | 2 |  |
| 11945 | South | Cat | 2020-2024 | 2 | 4 |  |
| 11948 | South | Cat | 2020-2024 | 2 | 2 |  |
| 11951 | South | Cat | 2020-2024 | 2 | 2 |  |
| 11952 | South | Cat | 2020-2024 | 2 | 4 |  |
| 11969 | South | Cat | 2020-2024 | 4 | 2 |  |
| 11987 | South | Cat | 2020-2024 | >16* | 1 | 0.125 |
| 11988 | South | Cat | 2020-2024 | >16 | 2 | 0.25 |
| 11989 | South | Cat | 2020-2024 | 2 | 2 |  |
| 11993 | South | Cat | 2020-2024 | 2 | 2 |  |
| 11994 | South | Cat | 2020-2024 | 1 | 2 |  |
| 11995 | South | Cat | 2020-2024 | >16* | 4 | 0.125 |
| 11996 | South | Cat | 2020-2024 | >16* | 4 | 0.125 |
| 11997 | South | Cat | 2020-2024 | 2 | 2 |  |
| 11998 | South | Cat | 2020-2024 | 2 | 2 |  |
| 11999 | South | Dog | 2020-2024 | 4 | 2 |  |
| 12000 | South | Cat | 2020-2024 | 1 | 2 |  |
| 12004 | South | Cat | 2020-2024 | >16 | 2 | 0.25 |
| 12005 | South | Cat | 2020-2024 | >16* | 4 | 0.125 |
| 12007 | South | Dog | 2015-2019 | >16* | 2 | 0.125 |
| 12008 | South | Cat | 2015-2019 | >16* | 2 | 0.5 |
| 12009 | South | Cat | 2020-2024 | >16* | 2 | 0.125 |
| 12010 | South | Dog | 2020-2024 | 2 | 22 |  |
| 12064 | South | Cat | 2020-2024 | >16 | 2 | 0.5 |
| 12077 | South | Human | 2020-2024 | 2 | 2 |  |
| 12085 | South | Cat | 2020-2024 | 2 | 2 |  |
| 12125 | South | Cat | 2020-2024 | 1 | 2 |  |
| 12142 | South | Cat | 2020-2024 | 2 | 2 |  |
| 12143 | South | Cat | 2020-2024 | 2 | 2 |  |
| 12167 | South | Cat | 2020-2024 | >16* | 2 | 0.5 |
| 12170 | South | Cat | 2020-2024 | >16* | 2 | 0.125 |
| 12171 | South | Cat | 2020-2024 | 2 | 2 |  |
| 12197 | South | Cat | 2020-2024 | 1 | 2 |  |
| 12218 | Metropolitan | Human | 2020-2024 | 2 | 4 |  |
| 12221 | Metropolitan | Human | 2020-2024 | 4 | 8 |  |
| 12222 | Metropolitan | Human | 2020-2024 | 1 | 2 |  |
| 12223 | Metropolitan | Human | 2020-2024 | 2 | 4 |  |
| 12225 | Metropolitan | Human | 2020-2024 | >16* | 4 | 0.25 |
| 12226 | Metropolitan | Human | 2020-2024 | 1 | 2 |  |
| 12228 | South | Cat | 2020-2024 | 2 | 2 |  |
| 12230 | South | Cat | 2020-2024 | 2 | 2 |  |
| 12231 | South | Cat | 2020-2024 | >16* | 2 | 0.063 |
| 11672 | South | Cat | 2020-2024 | 1 | 1 |  |
| 10681 | South | Cat | 2020-2024 | 1 | 2 |  |
| 12241 | South | Human | 2020-2024 | >16* | 2 | 0.125 |
| 12245 | South | Human | 2020-2024 | >16* | 4 | 0.125 |
| 12264 | South | Human | 2020-2024 | 1 | 2 |  |
| 12285 | South | Human | 2020-2024 | 0.5 | 4 |  |
| 12304 | South | Human | 2020-2024 | 1 | 2 |  |
| 12507 | South | Human | 2020-2024 | 2 | 2 |  |
| 12592 | South | Human | 2020-2024 | 1 | 1 |  |
| 12583 | South | Human | 2020-2024 | >16 | 4 | 0.063 |
| 12584 | South | Human | 2020-2024 | >16 | 2 | 0.5 |
| 12400 | South | Cat | 2020-2024 | 1 | 2 |  |
| 12409 | South | Cat | 2020-2024 | 1 | 2 |  |
| 12410 | South | Cat | 2020-2024 | 2 | 2 |  |
| 12250 | South | Cat | 2020-2024 | 4 | 4 |  |
| 12294 | South | Cat | 2020-2024 | 2 | 2 |  |
| 12717 | South | Human | 2020-2024 | 1 | 2 |  |
| 12823 | South | Human | 2020-2024 | 0.5 | 2 |  |
| 13015 | North | Human | 2020-2024 | 2 | 8 |  |
| 13016 | North | Human | 2020-2024 | >16* | 4 | 0.25 |
| 13017 | Metropolitan | Cat | 2020-2024 | 16 | 2 | 0.008 |
| 13018 | Metropolitan | Cat | 2020-2024 | >16 | 2 | 0.25 |
| 13019 | Metropolitan | Cat | 2020-2024 | >16 | 2 | 0.25 |
| 13020 | Metropolitan | Cat | 2020-2024 | 2 | 8 |  |
| 13022 | Metropolitan | Cat | 2020-2024 | 1 | 8 |  |
| 13023 | Metropolitan | Cat | 2020-2024 | 1 | 8 |  |
| 13024 | Metropolitan | Cat | 2020-2024 | 4 | 8 |  |
| 13025 | Metropolitan | Cat | 2020-2024 | 0.5 | 8 |  |
| 13026 | Metropolitan | Cat | 2020-2024 | 0.5 | 8 |  |
| 13027 | Metropolitan | Cat | 2020-2024 | 1 | 8 |  |
| 13029 | Metropolitan | Cat | 2020-2024 | >16* | 2 | 0.25 |
| 13030 | Metropolitan | Cat | 2020-2024 | 1 | 8 |  |
| 13031 | Metropolitan | Cat | 2020-2024 | 2 | 4 |  |
| 13033 | Metropolitan | Cat | 2020-2024 | 1 | 8 |  |
| 13034 | Metropolitan | Cat | 2020-2024 | 1 | 8 |  |
| 13035 | Metropolitan | Cat | 2020-2024 | 1 | 8 |  |
| 13036 | Metropolitan | Cat | 2020-2024 | 2 | 4 |  |
| 13037 | Metropolitan | Cat | 2020-2024 | 2 | 8 |  |
| 13038 | Metropolitan | Cat | 2020-2024 | 0.5 | 8 |  |
| 13039 | Metropolitan | Cat | 2020-2024 | 2 | 4 |  |

Legend: ITRA: Itraconazole; AMB: Amphotericin B; TER: Terbinafine; * indicates reduced growth compared to the positive control.
