# Supplementary material for: Unique Genetic Clades and Frequent Zoonotic Transmission of Sporothrix brasiliensis with Reduced Itraconazole Susceptibility
Source: Mycopathologia. 2026 Sep 15;191(5):80. doi: 10.1007/s11046-026-01101-5 (PMC13578069; doi:10.1007/s11046-026-01101-5)
Supplement: Supplementary file 2 [file 11046_2026_1101_MOESM2_ESM.docx]

**Supplementary Figure S1:** Distribution of minimum inhibitory concentrations (MICs) for azoles against 450 *Sporothrix brasiliensis* isolates from Rio Grande do Sul, Brazil. Antifungal susceptibility testing was performed using the CLSI M38-A3 broth microdilution protocol. All isolates were tested in the mycelial phase, and MICs were determined after 72 h of incubation at 30 °C. Geometric mean (GM), MIC₅₀, and MIC₉₀ values are presented. (A) Posaconazole (0.016–16 µg/mL); (B) Voriconazole (0.016–16 µg/mL); (C) Isavuconazole (0.016–16 µg/mL); (D) Fluconazole (0.063–64 µg/mL).
