# Supplementary material for: Unique Genetic Clades and Frequent Zoonotic Transmission of Sporothrix brasiliensis with Reduced Itraconazole Susceptibility
Source: Mycopathologia. 2026 Sep 15;191(5):80. doi: 10.1007/s11046-026-01101-5 (PMC13578069; doi:10.1007/s11046-026-01101-5)
Supplement: Supplementary file 3 [file 11046_2026_1101_MOESM3_ESM.docx]

**Supplementary Figure S2:** Distribution of Sporothrix brasiliensis isolates from Rio Grande do Sul according to itraconazole susceptibility profiles by genotype clade; **(A)** proportion of WT and NWT isolates (p< 0.001); **(B)** proportion NWT isolates, distribution between unaffected growth and inhibited growth (p< 0.001).
